# Exploring potential associations between the human microbiota and reservoir of latent HIV

**DOI:** 10.1186/s12977-024-00655-w

**Published:** 2024-11-29

**Authors:** Nel Marín-Sánchez, Roger Paredes, Alessandra Borgognone

**Affiliations:** 1 IrsiCaixa, Badalona, Catalonia Spain; 2 Universitat de Barcelona, Barcelona, Catalonia Spain; 3 Department of Infectious Diseases, Hospital Germans Trias i Pujol, Badalona, Catalonia Spain; 4 Department of Pathology, Center for Global Health and Diseases, Case Western Reserve University School of Medicine, Cleveland, OH USA

**Keywords:** HIV reservoir, Human microbiome, Bacterial inflammatory mediators, Microbial byproducts, Microbiome-based therapies

## Abstract

**Background:**

The rapid establishment and persistence of latent HIV-1 reservoirs is one of the main obstacles towards an HIV cure. While antiretroviral therapy supresses viral replication, it does not eradicate the latent reservoir of HIV-1-infected cells. Recent evidence suggests that the human microbiome, particularly the gut microbiome, may have the potential to modulate the HIV-1 reservoir. However, literature is limited and the exact mechanisms underlying the role of the microbiome in HIV immunity and potential regulation of the viral reservoir remain poorly understood.

**Results:**

Here, we review updated knowledge on the associations between the human microbiome and HIV reservoir across different anatomical sites, including the gut, the lungs and blood. We provide an overview of the predominant taxa associated with prominent microbiome changes in the context of HIV infection. Based on the current evidence, we summarize the main study findings, with specific focus on consistent bacterial and related byproduct associations. Specifically, we address the contribution of immune activation and inflammatory signatures on HIV-1 persistence. Furthermore, we discuss possible scenarios by which bacterial-associated inflammatory mediators, related metabolites and host immune signatures may modulate the HIV reservoir size. Finally, we speculate on potential implications of microbiome-based therapeutics for future HIV-1 cure strategies, highlighting challenges and limitations inherent in this research field.

**Conclusions:**

Despite recent advances, this review underscores the need for further research to deepen the understanding of the complex interplay between the human microbiome and HIV reservoir. Further integrative multi-omics assessments and functional studies are crucial to test the outlined hypothesis and to identify potential therapeutic targets ultimately able to achieve an effective cure for HIV.

## Introduction

One of the main challenges in developing an HIV cure lies in the rapid establishment and persistence of a viral reservoir following HIV-1 infection. With every replication cycle, HIV-1 integrates its proviral genome into the genome of host target cells. While this usually leads to cell death, a small fraction of target cells enters a long-term latency state [1]. Antiretroviral therapy (ART) suppresses HIV-1 replication in peripheral blood and in most tissues. This leads to increases in CD4 + T-cell counts, qualitative immune recovery and increased life expectancy. However, ART is not curative. Although antiretrovirals inhibit viral replication, they are unable to clear latent HIV-infected cells [2]. The reservoir mostly, but not exclusively, consists of latent CD4 + T-cells that have the potential to produce HIV-1 RNA, proteins and virions when no antiretroviral treatment is administered and they become activated. In addition, the latent reservoir is maintained by clonal expansion of HIV-1 infected cells through multiple proliferation mechanisms [3]. Hence, different HIV cure approaches [4, 5], including strategies aimed at eliminating the HIV reservoir, are being explored (Table 1).

**Table 1:** Current strategies in HIV cure research

HIV cure strategies	Approaches	Description	References
Direct reservoir manipulation	Latency reversal	Induction of the HIV provirus transcription in order to be targeted (“shock and kill”)	[6–8]
	Latency silencing	Permanently silencing of the integrated provirus (“block and lock”)	[9, 10]
	Gene editing	In vivo disruption of proviruses using gene editing (CRISPR technologies)	[11–15]
Immune modulation	Therapeutic vaccines	Boosting the immune system to eliminate HIV-infected cells	[16, 17]
	Neutralising antibodies	Target virions and induce antibody-dependent cytotoxicity	[18]
	Immune checkpoint blockade (ICB)	Inhibition of the binding of immune checkpoint proteins to revert T-cell exhaustion	[19]
	TLR-agonists	Boosting of innate & adaptive immunity	[20, 21]
Cell therapy	CAR-T cell	Engineered immune cells to target and kill HIV-infected cells	[13, 14]
	Cell therapies	Transplantation of HIV-resistant (CCR5-defective) immune cells	[22]

The gut-associated lymphoid tissue (GALT), in which reside most of the body lymphocytes [23, 24], has been increasingly proposed as a key anatomical target for HIV cure. Acute HIV-1 infection depletes gut CD4 + T-cells and disrupts the intestinal epithelial barrier integrity, which, in turn, promotes microbial translocation into the systemic circulation [25]. Gut microbiome dysbiosis - i.e., the imbalance in the composition and functions of the complex gut microbial community - contributes to perpetuate inflammation and immune activation in people with HIV (PWH) [26]. The establishment and persistence of other HIV reservoirs besides the GALT, such as the genital tract [27] and the lung lymphoid tissues [28, 29], may also be influenced by local microbiomes.

Observations suggesting an impact of the gut microbiome in the restoration of immune functions in PWH include: (a) changes in the gut microbial richness and diversity following HIV-1 infection, (b) similarities in the gut microbiome composition of elite controllers and HIV-uninfected individuals and (c) associations between CD4 + recovery and specific gut microbiota patterns [25, 30]. In this context, metabolic byproducts and microbial components have been shown to play a key role in shaping innate and adaptive immunity [31]. For instance, a recent study using germ-free compared to conventional humanized mice demonstrated that the resident microbiota significantly increase HIV acquisition and replication, and regulate levels of HIV target cells [32].

However, insights into the potential interplay between the microbiota and the HIV reservoir remain limited. This review will present most recently available evidence on the associations between human microbiome patterns and HIV reservoir, also exploring potential mechanisms behind such potential interaction. Implications and challenges for future treatment and cure strategies are also discussed.

## Microbiota changes associated with HIV infection

While multiple body niches - including the vaginal tract, respiratory tract and the oral cavity – have been colonized by microbial communities through evolution, the gut represents the largest and most diverse reservoir of microbes in the human body [33]. In healthy adults, the dominant gut microbial phyla *Bacteroidota* and *Bacillota* (formerly known as *Bacteroidetes* and *Firmicutes*) represent approximately 90% of the total composition, followed by *Actinomycetota*, *Fusobacteriota*, *Pseudomonadota*, *Verrucomicrobiota* and *Cyanobacteriota* (former *Actinobacteria*,* Fusobacteria*,* Proteobacteria*,* Verrucomicrobia* and *Cyanobacteriota*) [34, 35].

Although several studies have reported gut microbiome changes associated with HIV infection, comparing data across microbiome studies is challenging due to significant differences in sample collection, heterogeneity of the study cohort, technical aspects and uncertain impact of confounders, among other factors [36]. According to a number of meta-analyses attempting to integrate common findings [37–39], some of the most relevant compositional changes in the gut microbiome of PWH include:

- eduction in microbial gene richness [40] and changes in alpha diversity correlated with increased inflammation biomarkers [35];
- Depletion in methanogenic archaea [40] and butyrate-producing bacteria, mostly belonging to the class *Clostridia*, including *Ruminococcaceae* and *Lachnospiraceae* [41, 42];
- Increase of other bacterial species of the phylum *Bacillota*, including *Negativicutes*, *Bacilli* and *Erysipelotrichia* [41];
- Increased abundance of potentially pathogenic bacteria, particularly inflammatory Gram-negative bacteria such as *Pseudomonadota* [43], including *Enterobacteriaceae*, responsible for encoding genes involved in the metabolism of reactive oxygen and nitrogen species (ROS/RNS) [44];
- Compositional shift from *Bacteroides* to *Prevotella* dominance within the *Bacteroidota* phylum [45, 46].

It is worthy to notice that apart from the HIV status, several factors have been uncovered as a major source of microbiota variation, prior potential microbiota alterations linked to HIV infection. These include genetics, ethnicity, diet, age, sexual behaviours, antibiotic usage and disease status [47–49]. Factors such as sexual behaviour, demographics and ART regimen have emerged as major confounders in HIV-microbiota relationship studies (Table 2). Various studies have shown that *Prevotella*-enriched microbiomes are frequently observed in men who have sex with men (MSM) regardless of their HIV status, however driving factors and health implications for such profiles remain unknown [50, 51]. These microbiomes are also characterized by a greater microbial diversity compared to men who have sex with women (MSW) and individuals who acquired HIV through other transmission mechanisms.

Geographical and population-specific differences have also been observed in gut microbiome composition. In a recent study, distinct fecal microbiota profiles were observed in three different cohorts with different demographic and socioeconomics characteristics (US, Botswana and Uganda) [52]. *Bacteroides*-rich/*Prevotella*-poor microbiomes were associated with Western populations, as demonstrated by the higher representation of *Bacteroides* species in the US cohort, while *Prevotella* was more abundant in the Ugandan cohort. *Bacteroides*-rich/*Prevotella*-poor microbiomes have also been reported in PWH living in urban areas of Zimbabwe in contrast to more rural populations [53].

Age and ART usage are other well-known factors modulating the composition and diversity of the gut microbiome. Age-associated dysbiosis, often marked by reduced microbial diversity and shifts in specific bacterial populations including an increase in facultative anaerobes and a decrease in obligate anaerobes, has been found to closely resemble the dysbiosis observed in PWH [54]. However, only few studies have directly investigated the impact of age on the gut microbiomes of PWH. Regarding ART usage, although long-term ART has been associated with gut microbiome profiles in PWH resembling that of HIV uninfected people [26, 55], the specific impact of antiretrovirals on the gut microbiome of PWH remains to be deciphered.

**Table 2:** Main confounding factors in the gut microbiome in the context of HIV

Confounding factors	Characteristics	Main impact	References
Sexual practices	MSM	Increase in the species diversity and relative abundance of *Prevotella* while a depletion in *Bacteroides* is reported	[50, 51, 56]
Geography & culture	Demographics & urbanization	*Bacteroides*-rich/*Prevotella*-poor microbiome has been described as a “Westernization” of the microbiome with urbanization	[52, 53, 57]
Age	Age-associated dysbiosis	Increase in facultative anaerobes with inflammatory properties and a reduction in obligate anaerobes responsible for maintaining intestinal homeostasis	[54, 58, 59]
Art	Long-term ART regimen	The gut microbiome of long-term ART-treated individuals resemble the microbiome of HIV uninfected individuals	[26, 55, 60]

MSM = Men who have sex with men

## Current evidence on HIV reservoir-microbiota interplay

Only a few studies have specifically addressed the interplay between the human microbiome and HIV reservoir (Table 3). In the previous section, we have focused on the main microbial profiles described in HIV infection; here, we compile studies that examine associations between specific microbial patterns and direct measures of HIV reservoir size (HIV-1 DNA and HIV-1 RNA).

In a proof-of-concept, single-arm, therapeutic HIV vaccine study, the gut microbiome composition was linked to HIV-1 control during an analytical antiretroviral treatment interruption [61]. Borgognone et al. observed that viremic controllers (pVL < 2,000 copies/ml during 32 weeks of ART interruption, *n* = 3) had higher *Bacteroidales*/*Clostridiales* ratio and lower microbial gene richness compared to non-controllers (*n* = 10). The *Bacteroidales*/*Clostridiales* ratio negatively and significantly correlated with viral reservoir size (HIV-1 DNA and cell-associated HIV-1 RNA). Multi-omics integration analysis showed that *Bacteroidales* species, including *Bacteroides dorei* and *Bacteroides eggerthii*, positively correlated with immune activation transcripts and negatively with cellular HIV-1 DNA levels. Conversely, different species of *Clostridiales* such as *Subdoligranulum unclassified*, *Dorea formicigenerans* and *Eubacterium siraeum* showed the opposite pattern. Longitudinally, the gut microbiome of viremic controllers was consistently enriched in pro-inflammatory species including *Prevotella copri*, and depleted in methanogenic archaea and SCFAs-producing bacteria, such as *Roseburia intestinalis* and *Subdoligranulum ssp.*, typically associated with gut homeostasis preservation.

**Table 3:** Human microbiome and HIV reservoir association studies

Study population	Sample size	Main study findings	Microbiome sequencing method	Sample type	Microbiome associations with HIV reservoir size		References
					Positive	Negative	
Early-treated HIV+ patients	− Immune-mediated viremic controllers (n=3) − Non-controllers (n=10)	*Bacteroidales*/*Clostridiales* ratio as a novel gut microbiome signature associated with HIV-1 control	WGS	Gut (stool)	− *Subdoligranulum unclassified* − *Dorea formicigenerans* − *Eubacterium siraeum* Microbial gene richness	− *Bacteroides/Clostridiales* ratio − *Prevotella copri* − *Bacteroides dorei* *Bacteroides eggerthii*	[61]
HIV+ patients vs. uninfected controls	− ART-treated HIV+ patients (n=143) − Uninfected controls (n=190)	Microbial dysbiosis in PWH correlates with viral reservoir levels, cytokine production capacity and sexual behaviour	WGS	Gut (stool)	*Ruthenibacterium lactatiformans*	− *Firmicutes bacterium CAG 95* *Prevotella sp. CAG 5226*	[62]
HIV+ patients vs. uninfected controls	− ART-treated HIV+ patients (n=28) − Uninfected controls (n=9)	Association between higher levels of HIV-DNA in blood and reduced bacterial diversity in the lung microbiome of PWH	16S	Lung (BAL)	–	− Bacterial diversity (in PBMC) − *Prevotellaceae* − *Streptococcaceae Pasteurellaceae*	[28]
HIV+ patients (& classes) vs. uninfected controls	− ART-treated HIV+ patients (n=91) − TNs (n=30) − INRs (n=31) − IRs (n=30) − Uninfected controls (n=24)	Association of specific blood microbiota profiles with persistent inflammation and immune restoration in PWH	WGS	Peripheral blood & gut (stool)	− *Prevotella* spp. − *Porphyromonas gingivalis* − *Phocaeicola plebeius*	− *Burkholderia multivorans* − *Bacillus thuringiensis* − *Vibrio vulnificus* *Acinetobacter baumannii*	[63]

TNs = Treatment-naïve; INRs = Immunological ART non-responders; IRs = Immunological ART responders; WGS = Whole-Genome Sequencing, BAL = Bronchoalveolar lavage, PBMCs = Peripheralvblood mononuclear cells

In a second study, Zhang et al. compared gut microbiome data from 143 ART-treated PWH and 190 healthy individuals, describing strong correlations between microbial dysbiosis and viral reservoir levels, cytokine production capacity and sexual behaviour in PWH [62]. Inverse correlation between *Firmicutes bacterium* and *Prevotella* spp. with CD4 + T-cell-associated HIV-1 DNA and RNA, and a positive correlation between *Ruthenibacterium lactatiformans* and CD4 + T-cell-associated HIV-1 RNA were found. Consistent with previous findings [45, 50, 64], an increased abundance of *Prevotella* observed alongside depletion of *Bacteroides* and *Alistipes*, resulting in a significantly increased *Prevotella*/*Bacteroides* (P/B) ratio was observed in PWH. By comparing cytokine production between infected and uninfected individuals, Pam3Cys-induced IL-10 production was associated with *Prevotella copri*, while *Bacteroides vulgatus* associated with Pam3Cys-induced IL-1β production.

Two genetically different *P. copri* strains were identified. The strain enriched in HIV-negative controls positively associated with CD4 + T-cell levels and inversely with inflammatory cytokine production capacity compared to the PWH-related strain. On the other hand, the PWH-related strain associated with increased expression of IL-6 and IL-10, two previously described biomarkers linked to HIV pathogenesis [65].

Although the GALT is a major target of HIV infection and a reservoir for viral persistence, the pulmonary mucosa as an anatomical HIV reservoir and the associated microbiota have been also explored [66]. Wang et al. specifically investigated the interaction between the lung microbiome, pulmonary immunity and the HIV reservoir size; and how PWH could be predisposed to chronic lung disease [28]. Bronchoalveolar lavage (BAL) fluid samples from 28 ART-treated PWH and 9 healthy individuals were used to examine the local lung environment by 16 S rRNA sequencing. While smoking significantly decreased the Shannon diversity index, no difference in alpha diversity was observed between PWH and controls. Both smoking and HIV + status had an impact on lung bacterial community composition and increased within-group compositional variability. Although no relationships were found in BAL between lung microbial communities and peripherial HIV-1 reservoir, higher HIV-1 DNA levels in peripheral blood molecular cells (PBMCs) were associated with reduced bacterial diversity in the lungs of PWH under ART. Moreover, HIV-DNA levels in PBMC were also related to changes in lung bacteria community composition, including decreased abundance of *Prevotellaceae*, *Streptococcaceae* and *Pasteurellaceae*.

Microbial translocation from the gut to the systemic circulation is an observed feature of PWH, promoting chronic inflammation and immune activation [25]. Nevertheless, it is unclear which specific microbial groups in the blood are associated with HIV progression and immune recovery. Guo et al. investigated the impact of abnormal blood microbe profiles on inflammation and immune restoration in PWH compared to 24 healthy controls [63]. Stool samples were also collected to identify potential links between gut microbial translocation and disease pathogenesis.

While PWH showed reduced richness in stool samples compared to healthy controls, treatment-naïve individuals (TNs) displayed significantly higher alpha diversity of blood microbiota compared to healthy controls, immunological and non-immunological responders. These results suggested partial intestinal integrity and microbial translocation restitution achieved by ART. Moreover, increased abundance of *Bacteroidota* and *Bacillota* in the blood of TNs, compared to their decrease in stool samples, confirmed the hypothesis of bacterial translocation from the intestinal lumen to the systemic circulation. According to correlation analyses between differentially abundant blood microbial species and clinical indicators, enriched species in healthy controls - *Burkholderia multivorans*, *Bacillus thuringiensis*, *Vibrio vulnificus* and *Acinetobacter baumannii* - positively associated with CD4/CD8 ratio and CD4 + T-cell counts; whereas negative associations were observed with HIV DNA and RNA levels. Conversely, microbial species enriched in PWH, including *Prevotella* spp, *Porphyromonas gingivalis* and *Phocaeicola plebeius*, were negatively correlated with CD4 + T-cell counts and CD4/CD8 ratio, and positively correlated with HIV DNA and RNA.

## Potential key components mediating microbiome-HIV reservoir interactions

The microbiome and its metabolites play a crucial role in shaping and modulating both innate and adaptive immune systems [35]. Specifically, commensal bacteria can regulate the function of innate immune cells like macrophage and neutrophils [67] and stimulate the production of antimicrobial peptides and mucus by intestinal epithelial cells [68]. Also, the gut microbiota can influence B cell development and antibody production [67] and affect T cell differentiation, including the balance of Th1, Th2, and Treg cells, which are crucial for regulating immune tolerance and inflammation [69]. Additionally, microbial metabolites like short-chain fatty acids modulate immune responses both locally and systematically [68]. A number of microbiome studies in PWH have found associations between gut dysbiosis, microbial translocation, and increased inflammation and immune activation, suggesting that changes in the gut have significant impact in the pathogenesis and persistence of HIV infection [26]. Considering these interactions, it is plausible that such changes may impact the HIV reservoir in several ways, including chronic immune activation driven by microbial translocation, alterations in Treg populations impacting the control of HIV infected cells, metabolite-mediated effects influencing T cell functioning and alterations in mucosal immunity, among other potential mechanisms [70]. However, the exact mechanisms by which the human microbiome influences immune responses and how this may affect the HIV reservoir is still an emerging area of research.

Four studies describing associations between the human microbiome and the HIV reservoir have been reported in this review [28, 61–63]. Across different anatomical sites in PWH, specific microbial patterns have been associated with the viral reservoir size measured in distinct HIV contexts (Table 3). However, considering the limited evidence determining the exact dynamics of this potential interplay remains challenging. Despite these limitations, such findings may suggest a functional framework evidencing immune activation as a key determinant of HIV-related outcomes.

### Prevotella species and other commensal bacteria with inflammatory properties

In the studies presented in this review, *Prevotella* species have repeatedly emerged in the evaluation of microbiome-HIV reservoir interactions.

*Prevotella* is a diverse genus of Gram-negative bacteria with moderately saccharolytic capabilities and bile salt sensitivity [71]. Despite its abundance across multiple body sites, the role of this genus has increasingly come under the spotlight due to conflicting reports about whether its effect on human health is beneficial or detrimental [71]. When compared with other commensal bacteria, *Prevotella* exhibited increased inflammatory properties, as demonstrated by higher release of inflammatory mediators from immune and stromal cells [72].

In a number of studies, *Prevotella* enrichment was associated with intestinal inflammation, increased mucosal and systemic immune activation and impaired antiviral defences in PWH [73–75]. In untreated HIV-1-positive individuals, high expression of colonic myeloid dendritic cells positively correlated with HIV viral load and *P. copri* abundance, which in turn prompted the maturation of myeloid dendritic cells to produce inflammatory cytokines in vitro [76].

Another study integrating microbiome and metabolome profiles described *Prevotella* enrichment in the gut microbiota of elite controllers along with higher abundances of dipeptides tryptophylglycine and valylglutamine. These molecules showed an agonist effect on *Prevotella in vitro* as well as anti-HIV properties by binding to the HIV-1 envelope glycoprotein gp120 and inhibiting the entry into CD4 T-cells [77].

As reported earlier (Table 3), negative associations between *Prevotella* and the reservoir size were observed in the gut [61, 62] and pulmonary [28] microbiomes of PWH, which may be suggestive of a possible protective role against HIV-1 reservoir persistence. Whereas, positive associations between *Prevotella* in blood and the reservoir size [63] could be explained by increased microbial translocation in PWH. Despite the evidence discussed herein, due to its broad functional diversity, providing a conclusive interpretation on the exact biological role of *Prevotella* species in the maintenance of HIV reservoirs remains challenging [78].

Although a shift from *Bacteroides* to *Prevotella* has been described in PWH [45, 50, 64], other bacteria within the phylum *Bacteroidales* may also contribute to the establishment of a pro-inflammatory environment and potentially modulate the HIV reservoir. For instance, negative correlations between *Bacteroides spp* (*B. dorei* and *B. eggerthii*) and CD4 + T cell-associated HIV-1 DNA were reported in a study in which immune-mediated viremic controllers showed higher *Bacteroidales/Clostridiales* ratio [61]. Albeit no direct correlations with the viral reservoir were performed, in line with these findings, enrichment in *Bacteroidota* (*Bacteroides* and *Prevotella*) and pathways involved in host defense, such as acute inflammatory response to antigenic stimulus, type I interferon signaling and positive regulation of host immune response, were described in vaccine responders receiving dendritic cells-based HIV-1 immunization [79].

Collectively, these findings suggest a possible role of commensal bacteria with inflammatory properties in HIV viral control, although further insights to elucidate causal relationships are needed.

It is worth mentioning that the interpretation of ratio of different bacterial taxa (i.e. *Bacteroidales*/*Clostridiales*) or broad microbial groups proposed as potential biomarkers in the context of small descriptive cohort-based studies have limitations and should be approached with caution. Although such findings may be a focal point in primary research, their biological relevance and mechanistic insights need to be further explored in larger validation and functional studies to achieve a more accurate understanding of their implication in HIV persistence.

### Microbial metabolites and byproducts

As widely described, short-chain fatty acids (SCFAs) (i.e. acetate, butyrate and propionate) are the main metabolites derived from gut commensal bacteria, contributing to the maintenance of gut barrier integrity and inflammation reduction through Treg differentiation and TGF-β secretion [31]. In particular, butyrate produced by bacterial anaerobic fermentation of dietary carbohydrates is known as a potent modulator of immune and inflammatory responses. Of note, most butyrate-producing bacteria belong to the *Bacillota* phylum, and are predominantly classified within the cluster *Clostridia* [80].

In the context of HIV, microbial translocation of gut bacteria or bacterial byproducts to the systemic circulation can promote chronic inflammation and both innate and adaptive immune activation in PWH [35]. Moreover, regulatory immune responses via Treg and TGF-β along with histone deacetylase inhibition activity can promote the persistence of latent infected cells and the HIV reservoir [81].

Intriguingly, the studies reviewed here (Borgognone et al., 2022 & Zhang et al., 2023) showed that positive associations between bacteria and larger HIV reservoir size, involve gut bacteria from the *Bacillota* phylum (i.e. higher *Clostridia* class in non-controllers [61]). In this preprint [82], the authors observed increased abundance of *Lactobacillaceae* (anti-inflammatory bacteria and member of the *Bacillota* phylum) and related metabolites (SCFAs and bile acids, BAs) in a subset of immune non-responders (senescent-INRs) which correlated with Treg frequencies and promoted in vitro HIV latency establishment. Collectively, these data suggest that bacteria with known anti-inflammatory properties, such as *Bacillota* members, might be associated with the magnitude of the HIV reservoir.

Although butyrate is a well-established modulator of the immune system, other microbial metabolites have also been thought as major regulators of immune system activation and inflammation [83]. Similarly to the intestinal SCFAs, secondary BAs – modified in the human gut by commensal bacteria including *Bacteroidota*, *Actinomycetota* and *Bacillota* [31]- contribute to Treg differentiation and are suggested to promote latently infected cells through secretion of cytokines like TGF-β [84]. Apart from their role as regulatory immune response mediators, previous studies [85, 86] have described the dual role (both promoting and inhibiting) of BAs as regulators of IFN signaling. Specifically, early type I IFN signaling promoted viral control in SIV-infected NHPs receiving IFN-α2a [85]. Reduction of latent virus levels following IFN-α blockade of ART-treated SIV-infected NHPs suggested a detrimental role for prolonged type I IFN signaling, with implications for maintenance of the HIV reservoir.

In addition to SCFAs and BAs, other microbial components and surface-associated molecules from both gram-positive and gram-negative bacteria may act as important modulators of the immune system. One of the most studied bacterial surface components is the glycolipid known as lipopolysaccharide (LPS), which is conserved across most gram-negative bacteria, including the dominant bacterial order in the healthy gut microbiota *Bacteroidales* [87]. LPS from *Bacteroidales spp* can activate innate immune responses, induce TLR4 signaling [87] and the production of proinflammatory cytokines by monocytes, macrophages, and neutrophils [88, 89], albeit to a lesser extent than LPS from other typical pro-inflammatory bacteria. In the context of HIV, circulating LPS resulting from HIV-induced microbial translocation has been shown to correlate with increased T-cell and dendritic activation [90] and elevated plasma inflammatory factors [91]. Capsular polysaccharide A (PSA), primarily studied from *B. fragilis*, can also induce pro- and anti-inflammatory effects in specific conditions and immune contexts, including increased secretion of TNFα, IL-6, and CXCL-10 consistent with a pro-inflammatory interferon-driven response [92]. Interestingly, in a murine model it has also been described that *B. fragilis* lipooligosaccharide (LOS), distinct from the classical LPS domain and much-studied LPS of *E. coli*, would be responsible to induce IFN-β expression through TLR4-TRIF signaling [93]. Altogether, such observations underscore the complex duality of such surface-associated bacterial components and the context-specific influence of commensal bacteria that can turn into distinct functional role, particularly in HIV infection.

### Cytokines and host immune transcriptional signatures

HIV infection and subsequent microbial dysbiosis induce immune cell dysfunction and increased inflammatory state in PWH. Such response is characterized by overproduction of pro-inflammatory cytokines (IL-1, IL-6, TNF-α, and IFN-γ) and concomitant decrease in anti-inflammatory cytokines (IL-4 and IL-10), leading to a chronic inflammation state when ART is not administered [35].

As described in this review, Guo et al. identified correlations between plasma inflammation proteins in PWH and clinical indicators, including the HIV reservoir [63]. From 92 plasma inflammation proteins, LAP TGF-β1 showed positive correlations with CD4 + T-cell counts and the CD4/CD8 ratio, and negative correlations with HIV DNA. Notably, LAP TGF-β1 is a multifunctional protein exerting anti-inflammatory effects in multiple processes [94].

Here too, Borgognone et al. observed lower levels of pro-inflammatory proteins in non-controllers, in which increased abundance of anti-inflammatory bacteria negatively correlated with the viral reservoir size. In addition, upregulated genes in viremic controllers compared to non-controllers (such as *MPO*, *DEFA1*, *DEFA4* and *ELANE*) were functionally enriched in immune activation processes and positively associated with *Bacteroidales* species, which in turn negatively associated with the HIV reservoir size [61]. In another study showing higher abundances of butyrate-producing bacteria in immune non-responders, decreased expression of inflammation, cell cycling and apoptosis gene sets associated with higher inducible HIV were found [82]. Despite this evidence, additional studies also suggested positive associations between the HIV reservoir size and anti-inflammatory host immune biomarkers [63, 95].

Additional studies seeking to decipher the observed associations between resident microbes, host immune signatures and HIV reservoir are needed to allow for causal inference and fully grasp their implications in HIV persistence.

### Potential impact of immune activation and inflammation mediators on the HIV reservoir

In this review, we have addressed updated evidence on the associations between the human microbiota and HIV reservoir and potential modulators involved in this interplay. Although the current knowledge on this topic is very limited, the studies reviewed here might suggest associations between the microbiome, related byproducts and immune activation, potentially influencing HIV reservoir size and persistence.

**Fig. 1:**
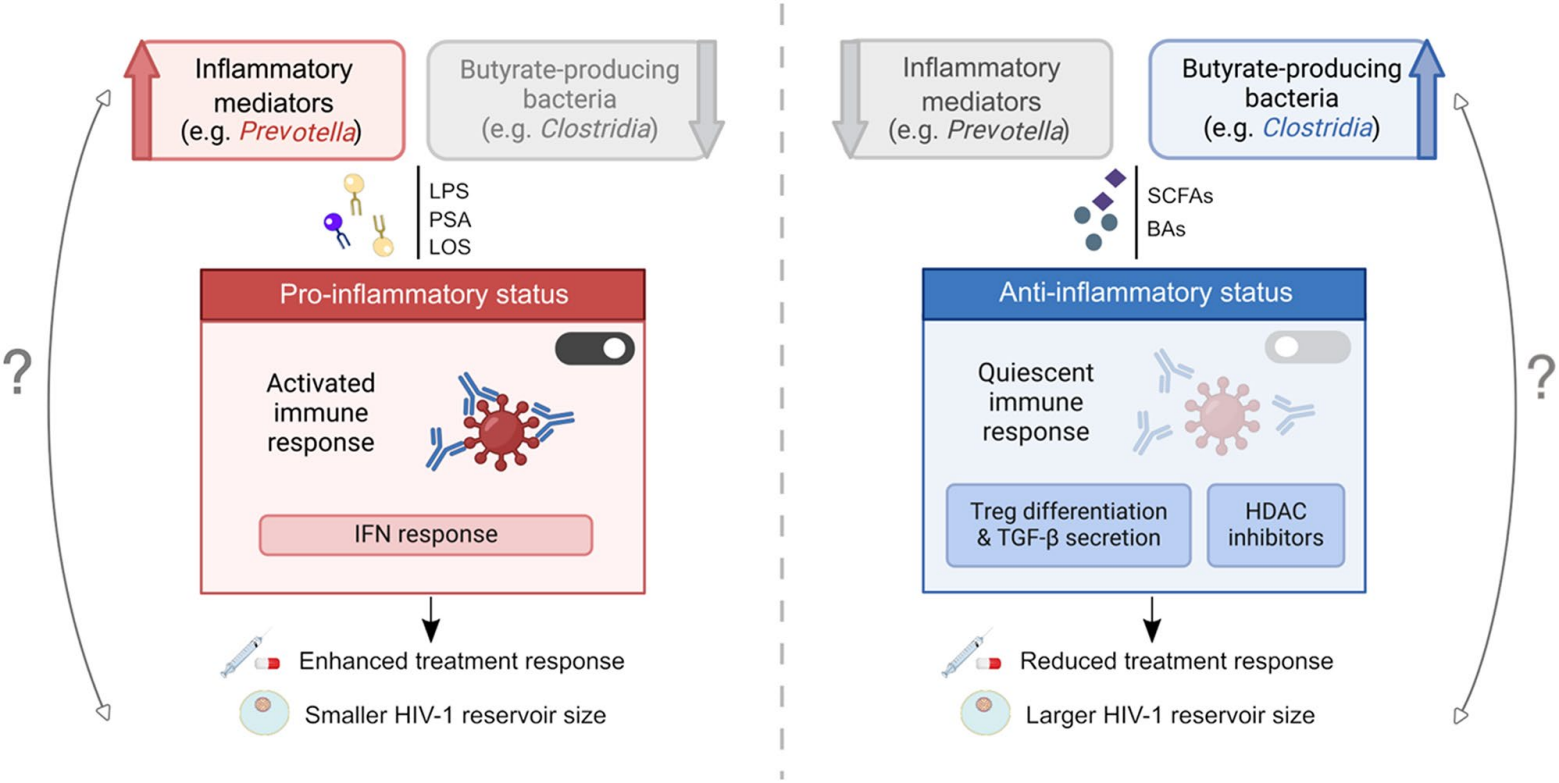
Hypothetical dynamics of bacteria modulation on HIV reservoir. Increased abundance and dissemination of bacterial-derived (i.e. *Prevotella* and *Bacteroides* species) inflammatory mediators, such as LPS, may induce host immune activation and acute inflammation, triggering prompt response to targeted HIV interventions, thus ultimately influencing the establishment of HIV-1 reservoirs. In contrast, anti-inflammatory bacteria, including butyrate producers, may promote an immune quiescent state and limit host immune response to specific treatments. Microbial metabolites, including SCFAs and BAs, may act as regulators of Treg differentiation, TGF-β secretion, and histone deacetylation inhibitors, modulating the latent HIV reservoir. Directionality and, by extension, causality in the proposed hypothetical framework underlying the interplay between HIV reservoir and microbiota remain uncertain. Abbreviations: BAs, bile acids; HDAC, histone deacetylase; HIV, human immunodeficiency virus; IFN, interferon; LPS, lipopolysaccharide; PSA, polysaccharide A; LOS, lipooligosaccharide; SCFAs, short-chain fatty acids; TGF-β, transforming growth factor beta. Figure generated with BioRender.com

We have discussed the contribution of inflammatory mediators to this interaction, particularly members of the *Prevotella* and *Bacteroides* genera, associated to smaller viral reservoirs. In this context, we speculate that bacterial products related to such genera, including LPS and PSA, might modulate IFN responses and act as mediators in the maintenance of the immune system activation [96] (Fig. 1). As an indirect evidence, findings discussed above have shown enrichment in anti-inflammatory bacteria (such as butyrate-producing) and decreased levels of inflammatory signatures in INRs, which in turn correlated with higher reservoir size. In this scenario, it is likely that microbial molecules, such as microbe-derived butyrate, might promote differentiation of Tregs and heighten the secretion of cytokines such as TGF-β [97, 98], contributing therefore to the maintenance of the latent HIV reservoir by promoting the persistence of latently infected quiescent cells [82, 99].

It is also likely that other SCFAs and BAs may promote the maintenance of latently infected cells through their function as HDAC inhibitors [100]. For example, β-hydroxybutyrate, a derivate of butyrate, can promote the acetylation of FOXO3A and downstream transcriptional programs that induce quiescent cells and thus maintenance of cells harbouring latent HIV [82, 101] (Fig. 1).

In summary, we hypothesize a functional framework in which basal acute inflammation and immune activation mediated by microbiome-related molecules may trigger prompt immune response to treatments and contribute to favourable outcomes. Conversely, a quiescent immune system state induced by anti-inflammatory mediators may limit host immune responses to HIV targeted treatments, thereby hindering the ability to reduce the reservoir size. While excessive inflammation can exhaust the immune system and impair treatment responses, in this context, moderate basal inflammation may enhance host immune responses to specific treatment aimed at reducing HIV persistence.

Although very preliminary and pending to further validation, these observations may provide new insights into the intricate functional dynamics underlying the immunomodulatory effect of resident bacterial communities on HIV persistence.

## Implications for future HIV cure strategies

Although suggestive associations exist, clearly defined contributions of the human microbiome to the HIV reservoir regulation remain poorly understood. Strategies aimed at modulating the microbiome in HIV cure efforts have focused on the interaction between the commensal bacteria (in particular from the gut microbiota) and related metabolites with systemic inflammation and immune activation [25].

Several studies have suggested the prominent role of microbial byproducts (metabolites and proteins) than the gut bacteria themselves in the modulation of inflammation and immune activation, and in turn of HIV reservoirs [102]. In this review, we have discussed the potential impact of bacteria-mediated inflammation on the reservoir size, exploring the immunomodulatory effects of bacterial components (LPS and PSA) and byproducts (SCFAs and BAs) in this functional framework. We speculate that, in this scenario, specific bacteria consortia or microbiome-derived molecules may work as adjuncts to functional cure strategies by modulating the host inflammation state and ultimately modulate the HIV reservoir size. This could be achieved through therapeutic approaches including prebiotics, probiotics, fecal microbiota transfer, dietary interventions and live biotherapeutic products (LBP) which have shown promise in reshaping the microbiota to modulate health outcomes in PWH [103]. For instance, engineered *Bacteroides* strains have been developed as probiotics and LBP for the treatment of inflammatory disorders [104, 105]. Nevertheless, despite recent advances, microbiome-related molecules are not yet established as robust clinical biomarkers or therapeutic targets in the context of HIV. Understanding the precise contributions and signalling mechanisms by which microbial molecules influence immune responses in PWH is pivotal for developing targeted next-generation therapeutics, especially in terms of timing, optimal composition and mode of delivery, to ultimately reduce HIV reservoirs.

## Limitations and challenges

In recent years, increasing efforts have been made to characterize how changes in the gut microbiota composition can affect the inflammatory processes in PWH and their impact on HIV persistence. Here, we have have sought to summarize updated knowledge on this field, highlighting possible functional frameworks. Nonetheless, this review faced notable limitations that need to be acknowledged. Firstly, we found that only four in silico and descriptive cohort studies directly explored the associations between the human microbiota patterns and the HIV reservoir. In these, microbiome samples were collected from distinct body niches (gut, lungs and blood), possibly contributing to inconsistent findings emerging when comparing studies.

Another intrinsic limitation was the small sample size and longitudinal sampling gaps, especially in pilot studies making it difficult to assess the reliability and robustness of results.

Moreover, different profiles within the HIV context were compared, - PWH vs. uninfected controls, viremic controllers vs. non-controllers or ART-responders vs. non-responders - adding another layer of complexity for identifying common patterns. This, coupled with substantial interindividual variability, generated by several endogenous and exogenous factors, complicated the identification of defined microbiota patterns for risk stratification. In addition, studies reviewed here were mainly based on omics techniques, and findings were largely correlative. In fact, insights presented are intented as a first step to establish associations and generate hypotheses. The next step is to decipher the nature of these interactions as mechanistic insight or experimental validation are still lacking. This could be achieved using comprehensive integrative multi-omics assessments (including microbiome, metabolic, host transcriptome and immune biomarker changes in the context of HIV) and longitudinal interventional studies followed by experimental validation. These integrated frameworks are necessary to shift the paradigm from exploring associational interactions to establishing causality in this research field. Moreover, further research into the feasibility of microbiome-based therapies in HIV is crucial to understand potential implications for disease progression and treatments.

## Conclusions

Direct evidence of the impact of the human microbiome on the HIV reservoir is very limited and causal mechanisms explaining their exact dynamics are lacking. A potential functional framework linking inflammatory microbial mediators with smaller HIV reservoir size has been outlined here, but important gaps in knowledge addressing the biological significance and generalizability of the observed associations remain. Further studies are required to elucidate the complex microbiome-immune system-HIV reservoir interplay, and to evaluate the ability of microbiome-based strategies to improve effective HIV remission and cure treatments.

## Data Availability

No datasets were generated or analysed during the current study.
